# Growth Stimulatory Effects and Genome-Wide Transcriptional Changes Produced by Protein Hydrolysates in Maize Seedlings

**DOI:** 10.3389/fpls.2017.00433

**Published:** 2017-03-30

**Authors:** Chiara Santi, Anita Zamboni, Zeno Varanini, Tiziana Pandolfini

**Affiliations:** Department of Biotechnology, University of VeronaVerona, Italy

**Keywords:** biostimulant, ionomic analysis, hormone metabolism, maize, microarray analysis, protein hydrolysates, root, transport

## Abstract

Protein hydrolysates are an emerging class of crop management products utilized for improving nutrient assimilation and mitigating crop stress. They generally consist of a mixture of peptides and free amino acids derived from the hydrolysis of plant or animal sources. The present work was aimed at studying the effects and the action mechanisms of a protein hydrolysate derived from animal residues on maize root growth and physiology in comparison with the effects induced by either free amino acids or inorganic N supply. The application of the protein hydrolysate caused a remarkable enhancement of root growth. In particular, in the protein hydrolysate-treated plants the length and surface area of lateral roots were about 7 and 1.5 times higher than in plants treated with inorganic N or free amino acids, respectively. The root growth promoting effect of the protein hydrolysate was associated with an increased root accumulation of K, Zn, Cu, and Mn when compared with inorganic N and amino acids treatments. A microarray analysis allowed to dissect the transcriptional changes induced by the different treatments demonstrating treatment-specific effects principally on cell wall organization, transport processes, stress responses and hormone metabolism.

## Introduction

The availability of mineral nutrients in the soil represents one of the most important limiting factors for crop productivity which is therefore highly dependent on the vast use of fertilizers (Tilman et al., 2002). However, excessive application of fertilizers as well as agrochemicals is causing severe environmental problems resulting in massive ecological degradation throughout the world (Tilman et al., 2002) Hence, the improvement of crop nutrient use efficiency that could reduce the use of fertilizers without any penalty on productivity, is worldwide an important goal (Baligar et al., 2001).

In this scenario, biostimulants are an emerging class of crop management products aiming at the mitigation of crop stress and improvement of nutrient assimilation (Halpern et al., 2015). The European Biostimulant Industry Council (EBIC) define these products as “materials which contain substance(s) and/or microorganisms whose function when applied to plants or the rhizosphere is to stimulate natural processes to benefit nutrient uptake, nutrient use efficiency, tolerance to abiotic stress, and/or crop quality, independently of its nutrient content” (http://www.biostimulants.eu). The emerging biostimulants market is estimated to grow of 10.4% from 2016 to 2021, reaching a value of 2.91 billion USD and an area of application of 24.9 million hectares by 2021 (http://www.marketsandmarkets.com/search.asp?Search=biostimulants).

A variety of biostimulant compounds are available in the market (reviewed in Calvo et al., 2014). They are classified as microbial inoculants, humic substances, fulvic acids, protein hydrolysates and amino acids, and seaweed extracts. These formulations are usually composed by different molecules and therefore their effect can be the result of many components that may work synergistically. The positive effects of biostimulants on plants include: yield increase (Ertani et al., 2009), increase of abiotic stress tolerance (Zhang et al., 2003; El Hadrami et al., 2010) and nutrient assimilation (Varanini and Pinton, 1995; Canellas et al., 2002), enhancement of fruit quality (Masny et al., 2004; Karakurt et al., 2009) and soil microbial activity (Chen et al., 2002). Their positive influence on plant growth is not due to a direct fertilization effect because they are active at very low concentration (Calvo et al., 2014). They indeed exhibit auxin-like and gibberellin-like activities and thus they are thought to function as signaling molecules (Ertani et al., 2009, 2013).

The protein hydrolysates have been proven to stimulate root growth and leaf biomass of several crops. du Jardin (2015) reviewed various effects resulting from the application of these compounds to crops. Direct effects on plants include modulation of N uptake and assimilation by regulation of enzymes involved in N metabolism and by acting on the signaling pathway of N acquisition in roots (Ertani et al., 2009, 2013). They can also regulate enzymes of the TCA cycle, contributing to the interplay of C and N metabolisms (Schiavon et al., 2008). Protein hydrolysates can improve plant antioxidant defense against free radicals thus mitigating environmental stress (du Jardin, 2015). They are also known to increase microbial biomass and activity, soil respiration and soil fertility (du Jardin, 2015). The application of protein hydrolysates can modify the morphology of the roots, facilitating nutrient uptake as a consequence of the increased absorptive surface area (Ertani et al., 2013). Moreover, the chelating and complexing activities of specific amino acids and peptides in the substrates are supposed to enhance nutrients availability and acquisition by roots (Colla et al., 2014).

In perspective of a circular economy, the use of protein hydrolysates can contribute to environment protection. Indeed, protein hydrolysates are generally produced from industrial and agricultural organic waste, turning them into high value-added products and, at the same time, reducing the costs derived from their disposal.

Although the effects of protein hydrolysates on crop performance have been documented, the scientific basis of their action has partially been elucidated mainly due to the complex nature of these products. The present work aims at shedding light on the effects and the action mechanisms of a commercial protein hydrolysates. The product used in this work is obtained by chemical hydrolysis of animal by-products and consists of a mixture of small peptides and a low percentage of free amino acids. We assessed the effects of the protein hydrolysates on maize root growth in comparison with the effects produced by an equal amount of N supplied either as a free amino acids mixture mimicking the biostimulant composition, or as N inorganic compound (NH_4_H_2_PO_4_). It is noteworthy that free amino acids are also included among the biostimulant compounds (Calvo et al., 2014). In order to elucidate the mechanisms underlying the effects observed in the root apparatus, root micro- and macro-nutrient accumulation was evaluated. Furthermore, we performed a transcriptome analysis that allowed identifying differential gene expression patterns in maize roots in response to the different forms of N supply highlighting global changes in gene transcription across multiple metabolic processes.

## Materials and methods

### Plant materials and growth conditions

Maize seeds (P0423 Hybrid, Pioneer Italia S.p.A.) were soaked in water for 24 h and germinated in the dark on wet filter paper for 72 h. The seedlings were then transferred to plastic pots containing 2 L of a 0.05 mM CaSO_4_ solution and grown for 24 h under a 16/8 h light/dark regime at 22–26°C, 40–50% relative humidity, 125 μE m^−2^s^−1^ light intensity. Each pot contained 12 seedlings that were grown in a diluted nutrient solution (Pinton et al., 1999) containing 100 mM MgSO_4_, 5 μM KCl, 200 μM K_2_SO_4_, 175 μM KH_2_PO_4_, 400 μM CaSO_4_, 25 μM NH_4_H_2_PO_4_, 2.5 μM H_3_BO_3_, 0.2 μM MnSO_4_, 0.2 μM ZnSO_4_, 0.05 μM CuSO_4_, 0.05 μM NaMoO_4_, 2 μM Fe-EDTA and supplemented with either protein hydrolysates (SICIT2000 S.p.A.) or inorganic nitrogen (NH_4_H_2_PO_4_) or a mixture of free amino acids mimicking the amino acids content of the protein hydrolysates (see also Results and Discussion section for treatment description). In all the treatments, the total N amount was kept constant at 5.65 or 11.3 mgL^−1^. After 3 days, roots of 24 seedlings for each treatment (protein hydrolysate, inorganic N and free amino acids) at 5.65 or 11.3 mgL^−1^ total N dose were collected for further analysis. The experiment was run three times obtaining three independent biological replicates.

### Phenotypic analysis of maize seedlings

Primary, seminal and lateral root average length was evaluated using ImageJ software. For the measurement of lateral roots length, the 10 longest roots per plant were considered. Primary, seminal and lateral root total length and surface area were measured with the aid of WinRHIZO™ scanner and automated software (Arsenault et al., 1995).

### Macro- and micro-nutrients quantification

The nitrogen concentration of the root samples was determined using the EA-IRMS Delta V (Thermo Fisher Scientific). The calibration curve for %N determination in dried tissues was performed using atropine (%*N* = 4.84).

Other macro- and micro-nutrients were quantified by ICP-MS analysis. Dried root samples (about 5 mg) were weighted and digested in a TFM microsampling insert using 250 μl of 69% ultrapure HNO_3_. Three inserts were put into 100-ml oven vessel containing 10 ml of water (milliQ, 18.2 M cm) and 1 ml of 30% H_2_O_2_. In addition, 5 mg of the following reference material were digested: NIST 1515 (apple leaves). Sample digestion was performed using a microwave oven (Milestone StartD® microwave). A 20-min ramping period was used to reach a digestion temperature of 180°C, which thereupon was maintained for 20 min. At the end, sample were diluted with water (milliQ, 18.2 M cm) to a final concentration of 3% HNO_3_. Multi-elemental analysis was carried out using the Agilent 7500cx ICP-MS (Agilent). The instrument was tuned using tuning solution (Agilent tuning solution 1 ppb) in a standard mode checking the sensitivity of masses ^7^Li, ^89^Y, and ^205^Tl and the oxide and double charged ion levels (<2%). Each macro- and micronutrient were quantified using a multi-element standard solution.

### RNA extraction and microarray analyses

Total RNA was isolated from plants treated with protein hydrolysates, inorganic N and free aminoacid mixture at the highest N concentration (11.3 mgL^−1^) using the Spectrum™ Plant Total RNA kit (Sigma-Aldrich) and quantified by spectrophotometry using NanoDrop™ 1000 (Thermo Scientific). RNA quality was evaluated using Agilent 2100 Bioanalyzer (Agilent). For each sample, the reactions of cRNA synthesis and labeling were carried out using 200 ng of total RNA and the Low Input Quick Amp Labeling Kit, One-Color (Agilent) and Cyanine 3 (Cy3)-CTP fluorescent dye according to the Agilent technical manual (http://www.agilent.com). Cy3-labeled cRNA (1.65 μg) of each sample was hybridized on a custom 4x44K Agilent array according to manufacturer's manual for 17 h at 65°C and scanned on Agilent G2565CA Microarray Scanner System (Agilent). Array hybridizations and washing were performed according to manufacturer's manual (One-Color Microarray-Based Gene Expression Analysis—Low Input Quick Amp Labeling—Protocol). Each “subarray” allow analyzing the expression of 39,372 maize transcripts predicted from the B73 reference genome (ftp://ftp.gramene.org/pub/gramene/maizesequence.org/release-5b/). Probe design was performed using Agilent eArray (http://www.genomics.agilent.com). Complete description of chip is available at the Gene Expression Omnibus (http://www.ncbi.nlm.nih.gov/geo) under the series entry (GPL22578). Feature intensities were extracted using Agilent's Feature Extraction Software 10.5.1.1 (Agilent). The hybridization data all samples were normalized using the value of the 75th percentile. Differentially expressed transcripts between Bio vs. N, Aa vs. N, and Bio vs. Aa were identified through Student's *t*-test using MeV software (http://mev.tm4.org/#/welcome) setting with the following parameters: *p*-value based on permutation with critical *p*-value of 0.01 and adjusted Bonferroni correction. Differentially expressed transcripts were filtered on the basis of fold changes value (|FC|≥2). All microarray expression data are available at the GEO (http://www.ncbi.nlm.nih.gov/geo) under the series entry (GSE89535).

### Quantitative RT-PCR analysis

For the quantitative RT-PCR (qRT-PCR) we used the same RNA samples extracted as described above. Three cDNA samples derived from 3 independent RNA samples were analyzed. DNase treatment and reverse transcription were performed as described in Molesini et al. (2014). cDNA amplification and PCR cycling conditions and product dissociation curve were also performed as indicated in Molesini et al. (2014). Data from qRT-PCR experiments were analyzed according to the 2^−ΔΔCt^ method. The list of primers adopted for qRT-PCR is reported in Supplementary Table 1. *UBCE* gene, coding for ubiquitin-conjugating enzyme, was used as reference gene (Manoli et al., 2012).

## Results and discussions

### Protein hydrolysates and free amino acids display different stimulatory effects on root growth

**Figure 1:**
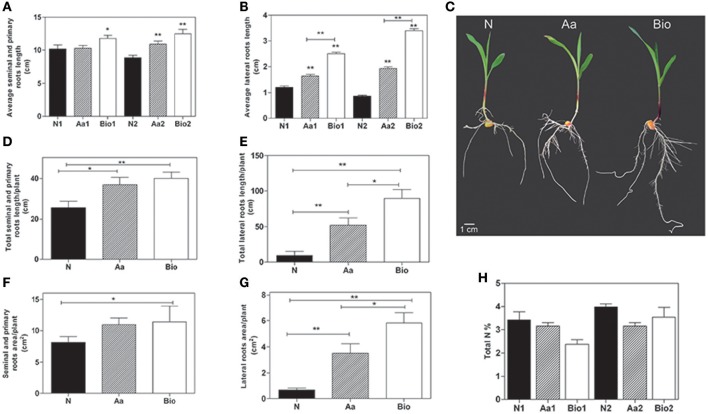
**Phenotypic analysis of maize roots after 3 days of treatment with inorganic nitrogen (N), amino acids (Aa), or protein hydrolysates (Bio)**. Average seminal and primary root length **(A)** and average lateral root length **(B)** of seedlings treated with protein hydrolysates (0.05 and 0.1 mlL^−1^) and seedlings treated with equivalent amounts of total N (5.65 and 11.3 mg L^−1^, respectively) supplied either as inorganic nitrogen (N) or as a mixture of free amino acids mimicking the composition in amino acids of the protein hydrolysate product. Root length was evaluated using ImageJ software (http://imagej.net). For lateral root length determination, the 10 longest roots were chosen manually. **(C)** Representative maize seedlings from N2, Aa2, and Bio2 treatments. Total length of seminal and primary roots **(D)**, total length of lateral roots **(E)**, total surface area of primary and seminal roots **(F)**, and total surface of lateral roots **(G)** of seedlings treated with a concentration of protein hydrolysates, free amino acids and inorganic nitrogen equal to11.3 mgL^−1^ of total N measured with WinRHIZO™ software. **(H)** Total N content in roots of seedlings treated with protein hydrolysates, free amino acids and inorganic N at two doses. In **(A,B,H)** N1, Aa1, and Bio1 refer to the lowest N dose (5.65 mg L^−1^) and N2, Aa2, and Bio2 refer to the highest one (11.3 mg L^−1)^. The average values are reported. Bars represent the standard error (SEM) [*n* = 24, except for data in **(H)**, where *n* = 3], if not otherwise specified, Student's *t*-test was applied vs. N-treated plants, ^*^P < 0.05; ^**^P < 0.01.

To investigate the effects of protein hydrolysates, we grew maize seedlings for 72 h after the emergence of the primary root in a N-free nutrient solution supplemented with the protein hydrolysate (Bio), inorganic nitrogen (NH_4_H_2_PO_4;_N) or a mixture of amino acids (Aa) mimicking the composition in amino acids of the protein hydrolysate. The protein hydrolysate is a liquid formulate derived from the hydrolysis of cow connective tissue, a by-product of tanning industry. It contains 30% (w/w) organic matters (C), 11.3% (w/w) total nitrogen (N), 10% (w/w) organic N, of which 62.5% (w/w) total amino acids and 10% (w/w) free amino acids (the detailed amino acids composition is summarized in Supplementary Table 2). The molecular weights of the peptides present in the protein hydrolysates range from 1,500 to 2,000 Da. To evaluate the dose response and the relative effects of Bio treatment on roots and shoots, we supplied the seedling with increasing doses of the biostimulant from 0.001 to 0.1 mlL^−1^. The growth of the shoots was not affected by the treatments, whereas the protein hydrolysate at 0.05 and 0.1 mlL^−1^ promoted the growth of the roots (Supplementary Figure 1). To analyze the effects of Bio treatments in terms of their contribution to N supply in the growth medium, we compared the root growth of maize seedlings treated with Bio at 0.05 and 0.1 mlL^−1^ and seedlings treated with equivalent amounts of total N (5.65 and 11.3 mgL^−1^, respectively) supplied either as inorganic N (NH_4_H_2_PO_4_) or free Aa. The Aa treatment consisted of a mixture of free amino acids identical in composition and concentration to the amino acids present in the protein hydrolysates described in Supplementary Table 2. Both Bio treatments induced root growth (Figures 1A–C), this effect was particularly evident for the lateral roots whose average length was approximately 2 and 3 times higher than that of seedlings supplied with 5.65 and 11.3 mgL^−1^ inorganic N, respectively. Also the Aa treatments showed the capacity to promote root growth compared to N. The effect was detectable in the primary and seminal roots at the higher Aa concentration, whereas the average length of lateral roots was increased also with the lower concentration (Figures 1A–C). Interestingly, the protein hydrolysates, containing only 10% of free amino acids, had always a stronger effect on root growth than a treatment consisting of free amino acids only.

A rapid and efficient growth of root apparatus can be advantageous during the first phases of seedling emergence after sowing, increasing the seedling capacity to absorb water and mineral elements (Lynch, 1995). Therefore, we calculated the total root length and area per plant of seedlings subjected to the different treatments using the WinRHIZO™ apparatus. The total length of both primary and seminal and lateral roots per plant was significantly higher in Bio (0.1 mlL^−1^) and Aa-treated seedlings than in those supplied with N (Figures 1D–G). The most striking effect was observed as expected, on total lateral root length. Considering the total surface area of the primary and seminal roots per plant, the highest value was measured in the Bio-treated seedlings, whereas the Aa treatment was ineffective (Figure 1F). The total surface area of the lateral roots increased by ~3 and 5-fold in Aa and Bio-treated seedlings, respectively as compared with seedlings treated with N (Figure 1G). Collectively, these results demonstrate the marked promoting effect of low doses of Bio on lateral root development; this effect is superior to that produced by free amino acids and inorganic N treatments equalized for N content.

To assess whether the effects of protein hydrolysate and free amino acids were due to increased N accumulation, we determined the total N concentration in roots of seedlings treated with Bio, N and Aa and N at two doses, corresponding to the addition of 5.65 and 11.3 mgL^−1^ N, respectively. The total N concentration in Bio-, Aa-, and N-treated roots did not differ (Figure 1H).

### Protein hydrolysates increase the uptake of specific nutrients

**Figure 2:**
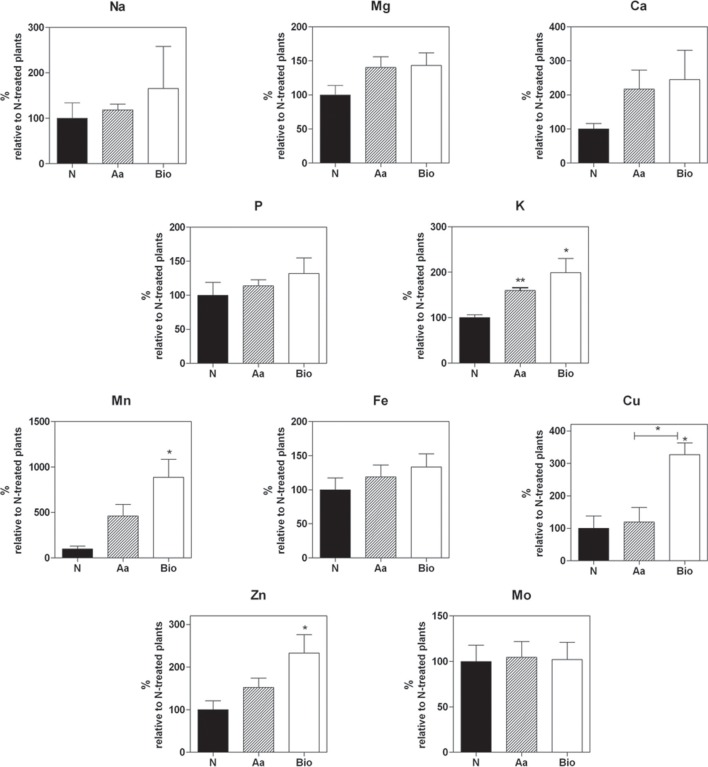
**Concentrations of macro- and micro-nutrients in the roots of seedling treated with protein hydrolysate, free amino acids and inorganic N**. Mg, K, Mn, Fe, Cu, Zn, Na, Ca, Mo, P root concentration was measured by means of high throughput inductively coupled plasma-mass spectroscopy (ICP-MS). In all the treatments, the total N supply was equal to 11.3 mgL^−1^. The nutrient concentrations are expressed as percentage of concentrations measured in seedlings treated with inorganic nitrogen. The average values are reported. Bars represent the standard error (SEM) (*n* = 3). If not otherwise specified, Student's *t*-test was applied vs. N-treated plants, ^*^P < 0.05; ^**^P < 0.01.

To study the effects of Bio and Aa on root nutrient accumulation, we quantified the macro- and micro-nutrient concentrations in the roots of seedlings treated with Bio, N, and Aa; in all the treatments, the total N supply was equal to 11.3 mgL^−1^ (Figure 2). The root concentration of Ca, Mg, Na, and P did not show statistically significant variations irrespectively from the treatment applied (Figure 2). Among the macronutrients, only K concentration was significantly increased in Bio- and Aa-treated seedlings compared with N-treated ones (Figure 2). Regarding the micronutrients, the concentration of Fe and Mo was not modified by the different treatments, whereas Cu, Mn, and Zn concentrations increased Bio-treated roots, but not in Aa-treated roots. The strongest effect of the Bio treatment was observed for Mn whose concentration was more than 8-fold higher in Bio-treated than in N-treated roots (Figure 2). The increased level of K in roots treated with organic N might be related to its function in maintaining ion balance and stabilizing cellular pH. The improved accumulation of Cu, Mn, and Zn in protein hydrolysate-treated roots might be the result of a specific action on metal trasporters (see Section Transport Processes) or the consequence of the peptide metal binding capacity that might facilitate nutrient availability.

### Global changes in the root transcriptome in response to protein hydrolysates and free amino acids

The transcriptional changes in maize roots subjected to 3-day treatment with Bio, N, or Aa were analyzed by means of genome-wide microarray hybridization analysis. For this aim, we used an Agilent chip that allowed to analyse the expression of 39,372 among the predicted maize transcripts (Schnable et al., 2009; Release 5b; http://www.maizesequence.org/index.html).

**Figure 3:**
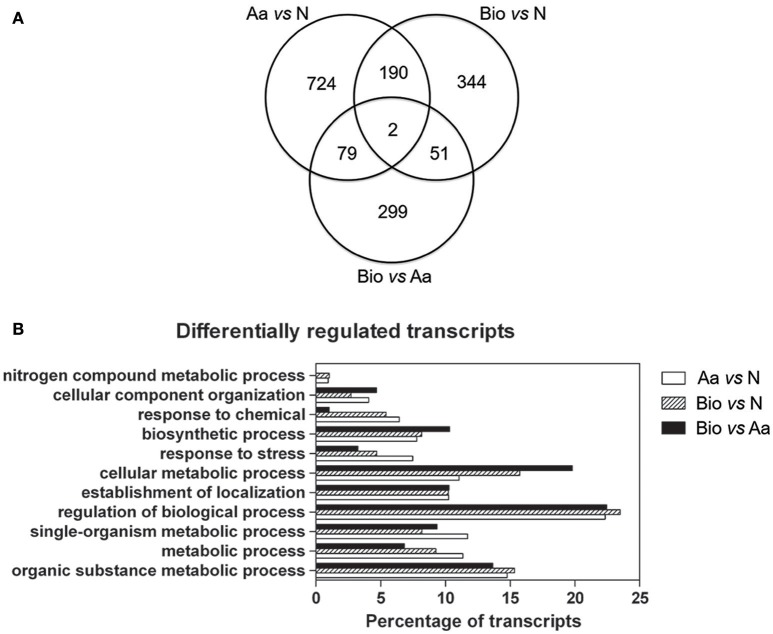
**Distribution of differentially regulated genes in the three comparisons and main functional categories of differentially expressed transcripts. (A)** Venn diagrams showing the shared and the specific differentially regulated transcripts in the different treatments (|FC| ≥ 2; adjusted *p*-value ≤ 0.01). **(B)** Distribution of differentially regulated transcripts in the three comparisons Bio vs. Aa, Bio vs. N and Aa vs. N grouped into main functional categories. For each functional category, the transcript percentage is calculated on the total of the differentially expressed transcripts minus those belonging to the “biological process” category.

Differentially expressed transcripts between roots supplied with different N forms were identified through a *t*-test (adjusted *p*-value ≤ 0.01 and |FC| ≥ 2). The analysis revealed that 995 transcripts were differentially expressed between Aa- and N-treated (Supplementary Table 3), 587 between Bio- and N-treated roots (Supplementary Table 4) and 431 between Bio- and Aa-treated roots (Supplementary Table 5) (Figure 3A), indicating high dissimilarity between the three transcriptional profiles. Moreover, 79 transcripts were differentially expressed both in the comparisons Aa vs. N and Bio vs. Aa, 51 were in common between Bio vs. Aa and Bio vs. N and 190 between Bio vs. N and Aa vs. N (Figure 3A) (Supplementary Tables S3–S5). Only two transcripts were differentially expressed in all the three comparisons (Supplementary Tables S3–S5). The transcriptional profile of 5 differentially expressed transcripts (*GRMZM2G347457_T01, GRMZM2G096958_T01, GRMZM2G429955_T01, GRMZM2G030036_T01*, and *GRMZM2G024996_T01*, coding respectively for peptide transporter, nicotianamine aminotransferase1, chlorophyll a-b binding protein 2, nicotianamine synthase 2, glycine-rich cell wall structural protein genes) was validated through qRT-PCR (Supplementary Figure 2). The annotation of all the up- and down-regulated transcripts was hand-curated, assigning them a “Gene Ontology” (GO) biological process term on the basis of a BlastP analysis. The transcripts were then grouped in main functional categories. In all the comparisons about 50% of the transcripts encode proteins with an unknown function and were assigned to the “biological process” class (Figure 3B, Supplementary Tables S3–S5). The other most abundant categories are “regulation of biological process,” “organic substance metabolic process” and “cellular metabolic process.” Interestingly, the transcripts belonging to “response to stress” were highly represented in Aa vs. N comparison while they were less abundant in Bio vs. Aa and Bio vs. N. Noticeably, the “nitrogen compound metabolic process” category was poorly represented, whereas the transcripts related to cellular transport (“establishment of localization”) were quite abundant.

#### Transcription factors

The “regulation of biological process” category included several transcripts encoding transcription factors (TFs). We identified 61 TF transcripts in the Bio vs. N, 35 in Bio vs. Aa and 89 in the Aa vs. N comparisons (Supplementary Tables S3–S5).

**Figure 4:**
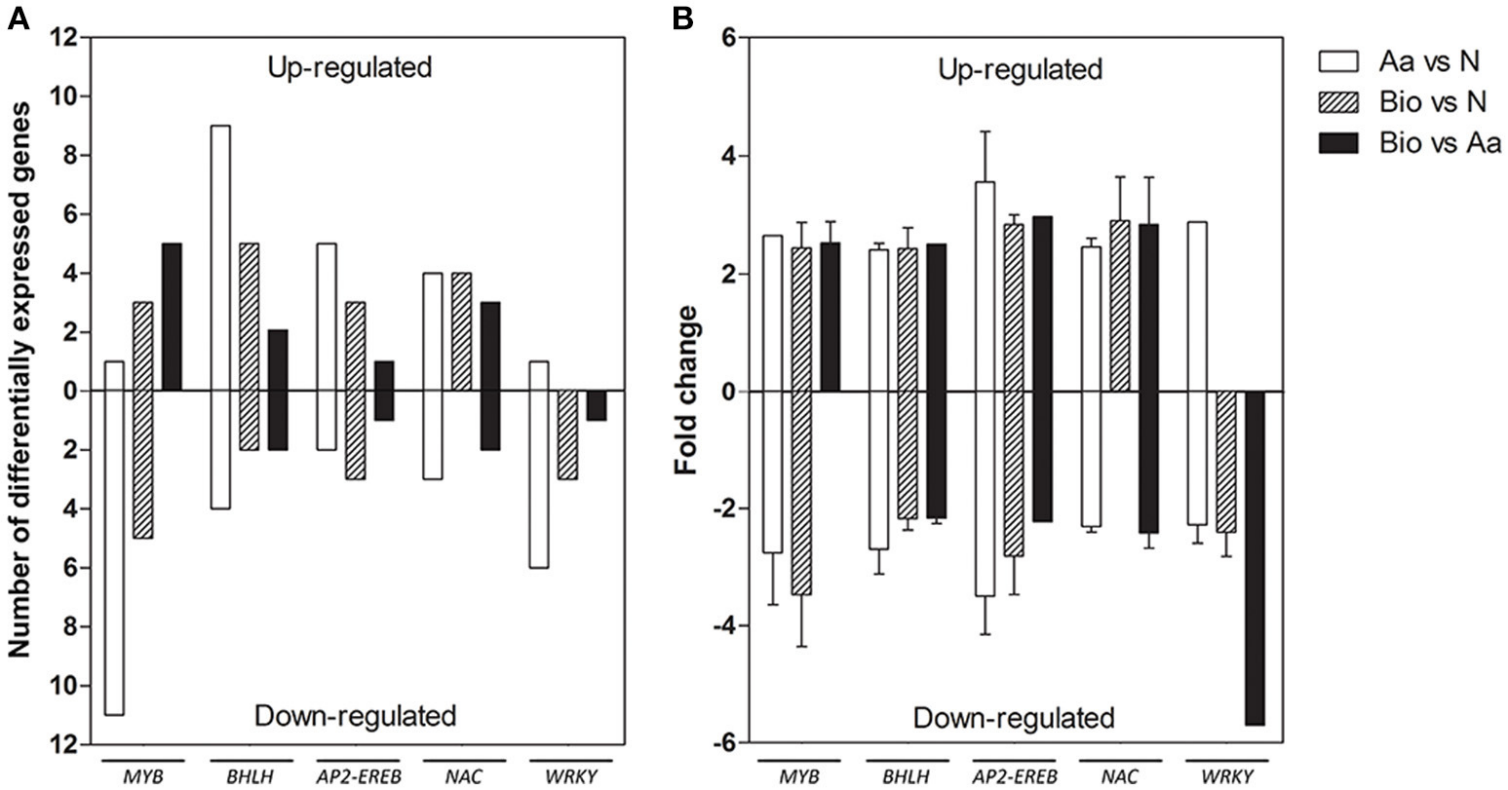
**Differentially expressed genes belonging to the most represented transcription factors families detected in the three comparisons, divided into up- and down-regulated. (A)** Number of differentially expressed genes. **(B)** Expression fold change. The data reported are means of FC values ± SD calculated for each transcription family except for those transcription factor families comprising a single transcript.

Concerning the distribution of these transcripts in TF gene families, *AP2-EREB, bHLH, MYB, WRKY, NAC* were the most represented families (Figure 4). Recently, *AP2-EREBP, bHLH, MYB*, and *WRKY* TF families have been shown to participate in the response to nutrient stress playing a major role in controlling regulatory network related to root development and N-deficiency response (Zhao et al., 2005; Rushton et al., 2012; Takehisa et al., 2013; He et al., 2016). Tai et al. (2016) also reported that members of some of these TF families were differentially expressed in primary, crown and seminal roots of maize, suggesting functional specialization of the different root types.

*AP2-EREB* transcripts were both up- and down-regulated in the different comparisons, whereas those encoding *WRKY*s were mostly down-regulated in all the three comparisons. A member of *AP2-EREB* family, preferentially expressed in coleoptile nodes during maize root development, plays a role in the formation of crown roots (Muthreich et al., 2013). *PLETHORA2* in Arabidopsis (*baby boom1*, the homolog in maize) encodes another AP2-EREBP TF required for the formation of root stem cells (Aida et al., 2004). *AP2-EREBP* and *WRKY* family were proven to be involved in the differential root response to N limitation of two Chinese maize inbred lines (Chen et al., 2015). *WRKY* transcripts were also induced in rice root after N, P, and K deficiency (Takehisa et al., 2013).

MYB proteins are important regulator of different physiological processes in plants, including development, metabolism and responses to environmental stresses (Li et al., 2015). In our work, a large number of *MYB* transcripts were modulated in response to the different N sources, resulting all up-regulated in the comparisons Bio vs. Aa, whereas in the comparison Aa vs. N, 11 members of this family were down-regulated. Some members of MYB family in *Arabidopsis* and rice regulate lateral root development and modulate auxin-mediated progression of lateral root development (Dai et al., 2012; Gibbs et al., 2014). MYB TFs can also respond to N deficiency; a member of this family in cucumber showed a rapid induction after N deprivation and functions in ethylene and auxin signaling (Zhao et al., 2015). Interestingly, these results well fit with the enhanced lateral growth in roots of Bio-treated seedlings (Figure 1C).

Gene family encoding bHLH TFs was largely up-regulated in all the three comparisons. This TF family is implicated in the control of various biological pathways, including the plant response to nutrient deprivation (Yang et al., 2016). A rice bHLH family member (OsPTF1) mediates tolerance to P deprivation in rice (Yi et al., 2005). Another member of this family plays an important role in adaptation to the P- and N-starvation in *Triticum aestivum* regulating genes involved in the uptake of P and N (Yang et al., 2016).

Several *NAC* transcripts were also differentially modulated in all three treatments. A variety of *NAC* members isolated from different species participate in both biotic and abiotic stress signaling pathways enhancing drought and salt stress tolerance (Liu et al., 2014; Su et al., 2015). Lu et al. (2015) analyzed the function of 7 maize NAC demonstrating their role in ABA-dependent abiotic stress responses. Interestingly, a NAC member of *Populus tremula x Populus alba*, is implicated in roots response to N deficiency, probably regulating root growth under low N conditions (Wei et al., 2013). Similarly, *NAC29* in *Arabidopsis* showed an elevated expression after N deprivation, and it was also responsive to chronic low N (Peng et al., 2007).

An interesting TF gene family is that of the LATERAL ORGAN BOUNDARIES domain (LBD) proteins. Intriguingly, every transcript belonging to this family was down-regulated in all the pairwise comparisons (*AC149818.2_FGT009* and *GRMZM2G092542_T01* in Bio vs. N; *GRMZM2G092483_T01* in Bio vs. Aa; *GRMZM2G092542_T01, AC234149.1_FGT002, AC149818.2_FGT009* and *GRMZM2G073044_T01* in Aa vs. N) (Supplementary Tables S3–S5). These proteins display high functional diversity including regulation of lateral root formation and N metabolism (Xu et al., 2016). In *Arabidopsis*, LBD16, LBD18, and LBD29 regulate lateral root organogenesis acting on auxin signaling pathway (Feng et al., 2012). The maize LBD protein RTCS (down-regulated in Aa vs. N and Bio vs. N) the closest homologs of AtLBD16/29, is involved in seminal and shoot-borne root initiation (Taramino et al., 2007). Moreover, lateral root emergence requires LDB-dependent activation of EXPANSIN (Kim and Lee, 2013). Members of the LBD family were shown to negatively regulate genes involved in response to N limitation in *Arabidopsis* (Rubin et al., 2009).

Finally, two members of TEOSINTE BRANCHED1/CYCOIDEA/PROLIFERATING CELL FACTOR (TCP) family were up-regulated in the comparisons Bio vs. N (*GRMZM2G180568_T01*) and Bio vs. Aa (*GRMZM2G060319_T01*). A member of this family (TCP20) expressed in root tips and vascular tissue of *Arabidopsis*, was shown to modulate lateral root growth in response to N supply and to regulate the nitrate transporter NRT1.1 expression (Guan et al., 2014).

Overall, this analysis may suggest that the different N sources produce different effects on root growth and metabolism by distinct modulation of TFs involved in the control of root development and N availability.

#### Cell wall components

In the present work, we found numerous differentially expressed genes belonging to “cellular component organization” category in all the pairwise comparisons (18 in Aa vs. N, 6 in Bio vs. N and 7 in Bio vs. Aa) (Table 1). Almost all the differentially expressed transcripts of this category encode extensins, expansins, pectinesterases, Casparian strip membrane proteins, xyloglucanendotransglucosylases/hydrolases, and glycine-rich cell wall structural proteins. In particular, the transcript *GRMZM2G024996_T01*, coding for a glycine-rich cell wall structural protein (GRP), showed the highest level of down-regulation in Bio vs. N (-19) and Aa vs. N (-63). This transcript showed high homology with a *Petunia hybrida* gene encoding GRP1 that is expressed during cell expansive growth and repressed during lignification (Condit, 1993). Genes involved in cell wall remodeling can regulate root growth and lateral root formation. Transcripts coding for expansins were modulated in all the three comparisons. It is well documented that these enzymes loosen the network of wall polysaccharides, allowing turgor-driven cell enlargement (Cosgrove, 2000). In addition, two transcripts for root Casparian strip proteins were up-regulated specifically in Aa vs. N. These proteins apparently regulate the transition of the lateral root primordia from flat to rounded morphology during root development (Lucas et al., 2013). Four and one transcripts coding for xyloglucanendotransglucosylases/hydrolases were differentially expressed in Aa vs. N and Bio vs. Aa, respectively. These enzymes play an important role in the remodeling of the xyloglucan/cellulose framework in the wall during cell growth and differentiation (Hara et al., 2014). Similarly, pectinesterase enzymes (modulated in Aa vs. N and Bio vs. N) are involved in the process of cell wall extension.

**Table 1:** **Differentially expressed transcripts involved in cell wall organization**.

**Genome ID**	**UniProt ID**	**Description**	**Aa vs. N (FC)**	**Bio vs. N (FC)**	**Bio vs. Aa (FC)**
GRMZM2G070913_T01	K7VZR1	Pectinesterase	3.77	2.81	
GRMZM2G112619_T01	K7U9U0	Xyloglucan endotransglucosylase/hydrolase	3.28		
GRMZM2G083888_T01	C5Y9U6	Casparian strip membrane protein 4	2.93		
GRMZM5G886185_T01	A0A096UH62	Xyloglucan endotransglucosylase/hydrolase	2.80		
GRMZM5G858456_T01	B6SRP0	Fucosyltransferase 7	2.80		
GRMZM2G455564_T01	K7TZQ3	Pectinesterase	2.41		
GRMZM2G127184_T01	B6SRP0	Fucosyltransferase 7	2.39		
GRMZM2G073079_T01	Q9ZT66	Endo-1,3;1,4-beta-D-glucanase	2.24		
GRMZM2G110832_T01	B6T959	Casparian strip membrane protein 1	2.23		
GRMZM2G144898_T01	Q1ZYQ8	Expansin-B10	2.00		
GRMZM2G114322_T01	Q8H274	Expansin-like A3	−2.02	−2.41	
GRMZM2G435380_T01	M8BPN6	Polygalacturonase	−2.06		
GRMZM2G168651_T01	P14918	Extensin	−2.11		
GRMZM5G870571_T01	M7ZVY5	Galactoside 2-alpha-L-fucosyltransferase	−2.16		
GRMZM2G392125_T01	A0A096TJQ7	Xyloglucan endotransglucosylase/hydrolase	−2.22		
GRMZM2G167637_T01	A0A096SUN3	Pectinesterase	−2.37		
GRMZM2G113761_T01	A0A096RUI5	Xyloglucan endotransglucosylase/hydrolase	−2.74		
GRMZM2G024996_T01	P27483	Glycine-rich cell wall structural protein	−63.07	−19.32	
GRMZM2G127072_T01	B6UAK6	Expansin-like 3		2.06	
GRMZM2G043943_T01	A0A096QHT7	Pectinesterase		−2.01	
GRMZM2G109842_T01	P35082	Profilin-2		−3.45	
GRMZM2G164785_T01	P0C1Y5	Expansin-B11			5.68
GRMZM2G152189_T01	B6STF8	Vegetative cell wall protein gp1			2.71
GRMZM2G153666_T01	B6UB39	Polygalacturonase			2.54
GRMZM2G413006_T01	A0A096TNC3	Xyloglucan endotransglucosylase/hydrolase			−2.19
GRMZM2G118759_T01	B6TEE0	Glycine-rich cell wall structural protein 2			−2.24
GRMZM2G469701_T01	M7ZJB5	Expansin-A22			−3.29
GRMZM5G870571_T01	K7V5L2	Uncharacterized protein			−3.58

*Genome ID, Maize transcript ID (ZmB73_5b_FGS_cdna.fasta.gz); FC, fold change value*.

Taken together these results suggest that transcriptional changes in genes encoding cell wall modifying enzymes induced by Bio and Aa, may results in cell wall remodeling that in turn affects root growth and architecture.

#### Stress-related transcripts

A relatively high number (38) of stress-related transcripts were differentially regulated in Aa vs. N comparison, whereas they were 14 and 6 in Bio vs. N and Bio vs. Aa, respectively (Table 2). About 50% of the stress related transcripts in Aa vs. N were represented by those coding for peroxidases (12 up-regulated, 5 down-regulated). At first glance, it is clear how the Aa treatment compared with N caused a higher stress response in the roots than Bio did. A possible explanation is that Aa caused an increase in ROS activity followed by an increase in expression of peroxidase genes for preventing H_2_O_2_ damage. Up-regulation of stress-related genes such as those for peroxidases and superoxide dismutases was observed in the root transcriptome of rice grown under P, K, and N deficiency (Takehisa et al., 2013). Moreover, ROS, such as H_2_O_2_ and ${\text{O}}_{2}^{-}$ are known secondary messages in several pathways associated with responses to biotic and abiotic stresses in plants (Apel and Hirt, 2004). Aside from their anti-oxidative activity, peroxidases are the most abundant enzymes in the cell wall where they display a multifunctional activity, also related to growth regulation (Vuletic et al., 2014). Peroxidases exist in multiple forms exhibiting different cellular localization and playing numerous biological functions. Therefore, to identify the specific involvement of peroxidases in the response of roots to Aa and Bio would require further investigation. Among the other differentially expressed genes in this category, the majority are transcripts involved in biotic stress response (Table 2).

**Table 2:** **Differentially expressed stress-related transcripts**.

**Genome ID**	**UniProt ID**	**Description**	**Aa vs. N (FC)**	**Bio vs. N (FC)**	**Bio vs. Aa (FC)**
GRMZM2G138450_T01	A0A096SAM6	Peroxidase	19.08		
GRMZM2G410175_T01	D7NLB3	Peroxidase	15.97		
GRMZM2G150731_T01	B4FYH1	Peroxidase	6.40		
GRMZM2G407740_T01	C0P3T3	Peroxidase	5.32		
GRMZM2G133475_T01	A5H454	Peroxidase 66	4.86		
GRMZM2G027217_T01	A0A096Q770	Peroxidase	4.76		
GRMZM2G010640_T01	A0A096PWP5	Peroxidase	4.27		
GRMZM2G404676_T01	A0A096TLS6	Peroxidase	4.19	2.11	
GRMZM2G023840_T01	K7VCN5	Peroxidase	3.46		
GRMZM2G009792_T01	A0A096PVZ5	Uncharacterized protein	3.41		
GRMZM2G037156_T01	K7UT08	Peroxidase	3.29		
GRMZM2G089982_T01	B6T3V1	Peroxidase	2.17		
GRMZM2G405459_T01	A0A096TLY3	Peroxidase	2.16		
GRMZM2G033665_T01	C5XUZ2	MLO-like protein	−2.06	−2.54	
GRMZM2G017116_T01	B6TR53	Defense-related protein	−2.07	−2.75	
GRMZM2G103342_T01	A0A096RME9	Peroxidase	−2.18		
GRMZM2G108847_T01	A3FMA3	Putative serine type endopeptidase inhibitor	−2.22	−2.01	
GRMZM2G112538_T01	Q29SB6	Pathogenesis-related protein 10	−2.33		
GRMZM2G112488_T01	D4HR93	Pathogenesis-related protein 10	−2.45		
GRMZM2G112524_T01	B6TR52	Pathogenesis-related protein 1	−2.52	−2.11	
GRMZM2G430500_T01	A0A096TRQ6	Uncharacterized protein	−2.60		
GRMZM2G374971_T01	P33679	Zeamatin	−2.61	−3.07	
AC197758.3_FGT004	K7U6B6	Peroxidase	−2.67		
GRMZM2G108207_T01	B4FH68	Peroxidase	−2.67		
GRMZM2G466563_T01	Q6YYA1	Putative calmodulin-binding protein	−2.72		
GRMZM2G117971_T01	A0A059Q1C7	Pathogenesis-related protein	−2.72		
GRMZM2G422240_T01	B4G197	16.9 kDa class I heat shock protein 1	−2.73		
GRMZM2G061766_T01	K7V347	Uncharacterized protein	−2.88		
GRMZM2G171078_T01	A0A096SWX6	Peroxidase	−2.89		
GRMZM5G899188_T01	Q08275	17.0 kDa class II heat shock protein	−3.36		
AC214360.3_FGT001	Q6Z5J6	Ent-pimara-8(14),15-diene synthase	−3.44		
GRMZM2G176085_T01	B4FYD8	Peroxidase	−3.46		
GRMZM2G158232_T01	B4G197	16.9 kDa class I heat shock protein 1	−3.49	−2.22	
GRMZM2G335242_T01	B6TQD6	17.4 kDa class I heat shock protein	−3.50		
GRMZM2G028306_T01	Q1EG72	(S)-beta-macrocarpene synthase	−3.78	−4.81	
GRMZM2G073510_T01	B6UFK1	Mating-type switching protein swi10	−3.79		
GRMZM2G419675_T01	B6TRF5	Win1	−4.10	−5.31	
GRMZM2G117989_T01	B6TRF5	Win1	−4.64		
GRMZM2G078013_T01	W0NT67	NBS-LRR disease resistance protein		2.45	
GRMZM2G037146_T01	Q8W0Q8	Small heat shock-like protein		2.14	
GRMZM2G168447_T01	M8B2N3	Pathogenesis-related protein 1A		−2.10	
GRMZM2G080183_T01	B4FVT4	Peroxidase		−3.14	
GRMZM2G117942_T01	A0A059Q1C7	Pathogenesis-related protein		−3.25	
GRMZM2G312597_T01	N1QRC0	Subtilisin inhibitor 1			2.55
GRMZM2G085934_T01	B6U175	17.5 kDa class II heat shock protein			2.23
GRMZM2G063438_T01	AAMT3	Anthranilate O-methyltransferase 3			2.12
GRMZM2G008740_T01	D9J101	Benzoate O-methyltransferase			−2.20
GRMZM2G080689_T01	K7VH54	Peroxidase			−2.39
GRMZM2G145552_T01	M7YYQ7	Cucumber peeling cupredoxin			−3.45

*Genome ID, Maize transcript ID (ZmB73_5b_FGS_cdna.fasta.gz); FC, fold change value*.

#### Transport processes

Differentially expressed transcripts grouped in “establishment of localization” are involved in several transport processes (Table 3). We focused on genes playing a role in transport processes of amino acids, peptides, ${\text{NO}}_{3}^{-}$ and ${\text{NH}}_{4}^{+}$. Considering amino acid transport, only in the Aa vs. N comparison, we found the up-regulation of a transcript encoding a putative amino acid permease (AAP; *GRMZM2G180547_T01*) that could be involved into transport across membranes. This result suggests that amino acid supply as N-source positively affected components involved in their uptake and/or translocation (Tegeder, 2012; Tegeder and Ward, 2012). Members of Amino Acid-Polyamine-Organocation (APC), Drug/Metabolite Transporter (DMT), ATP Binding Cassette (ABC), and Major Facilitator (MFS) super families play a role in amino acid export from the cytosol to apoplastic space or into intracellular compartments such as the vacuole (Okumoto and Pilot, 2011). Aa and Bio treatments modulated the expression of transcripts encoding protein belonging to DMT, ABC, and MFS families that can mediate these transport processes (Supplementary Tables S3, S4). In addition, members of the ABC and PTR families can be involved also in peptide transport (Koh et al., 2002; Waterworth and Bray, 2006). Other peptide transporters belong to the oligopeptide transporter (OPT) family (Koh et al., 2002; Waterworth and Bray, 2006). Concerning the PTR transporters, we observed a prevailing negative modulation of transcripts caused by Aa and Bio (*GRMZM2G026523_T01, GRMZM2G122712_T01, GRMZM2G347457_T01, GRMZM2G057611_T01, GRMZM2G015767_T01*). Focusing on OPT family, only the comparison Bio vs. N underlined the up-regulation of a transcripts encoding a putative transporter (*GRMZM2G152555_T01*) suggesting a role of this gene in transport of peptides in Bio-treated maize roots.

**Table 3:** **Differentially expressed transcripts involved in transport processes**.

**Genome ID**	**UniProt ID**	**Description**	**Aa vs. N (FC)**	**Bio vs. N (FC)**	**Bio vs. Aa (FC)**
GRMZM2G010251_T01	B4FSV9	High affinity nitrate transporter	9.37		
GRMZM2G156599_T01	Q9AY27	Iron-phytosiderophore transporter yellow stripe 1	8.60	2.25	
GRMZM2G000614_T01	Q7FMW4	ABC transporter G family member 38	3.94		
GRMZM2G135291_T01	G3XDL3	Putative iron-phytosiderophore transporter	3.82		
GRMZM2G180547_T01	Q53LH2	Amino acid carrier, putative, expressed	3.53		
GRMZM2G099382_T01	B6T0F4	Tonoplast dicarboxylate transporter	3.30		
GRMZM2G072071_T01	B6U4J2	ATP-binding cassette sub-family B member 10	2.76		
GRMZM2G148060_T01	K7VD92	Putative ferroportin-domain family protein	2.70		
GRMZM2G118507_T01	K7VD86	Uncharacterized protein	2.65		
GRMZM2G024196_T01	Q7XKF4	Probable metal-nicotianamine transporter YSL13	2.53	3.89	
GRMZM2G064437_T01	B6TDG1	Proton myo-inositol cotransporter	2.53		
GRMZM2G059465_T01	K7TWC7	Calcium-transporting ATPase	2.49		
GRMZM2G129843_T01	B6U7Q9	Lipid binding protein	2.41		
GRMZM2G362848_T01	V9SBV7	Nucleobase cation symporter 1	2.32		
GRMZM2G029951_T01	A0A096Q8Z7	Uncharacterized protein	2.29		
GRMZM2G123884_T01	Q7XVB3	Probable sodium/metabolite cotransporter BASS1, chloroplastic	2.21		
GRMZM2G056908_T01	Q9ATL8	Aquaporin TIP2-2	2.19		
GRMZM2G053991_T01	Q5W7C1	UPF0014 membrane protein STAR2	2.04		
GRMZM2G142924_T01	A0A096SDC7	Uncharacterized protein	−2.00		
GRMZM2G024808_T01	B6U7W3	Nitrate and chloride transporter	−2.02		
GRMZM2G072955_T01	M8CTF4	Chloride channel protein	−2.06	−2.02	
GRMZM2G153920_T01	B4FQN6	Sorbitol transporter	−2.07		
GRMZM2G137421_T01	B6TSV4	Peptide transporter PTR2	−2.09	−2.12	
GRMZM2G457523_T01	Q2QLJ1	Sodium/hydrogen exchanger family protein, expressed	−2.10		
GRMZM5G872392_T01	B6T9U6	Bidirectional sugar transporter SWEET	−2.15		
AC186166.3_FGT008	A0A096PGB1	Uncharacterized protein	−2.16		
GRMZM2G112456_T01	C0HIN0	Oligopeptide transporter 4	−2.17		
GRMZM2G047431_T01	Q69EY5	GDP dissociation inhibitor protein	−2.17		
GRMZM2G080178_T01	B6UC24	Sulfate transporter 1.2	−2.21		
GRMZM2G122712_T01	Q6AU97	Putative proton-dependent oligopeptide transporter (POT)	−2.26	−2.96	
GRMZM2G009344_T01	B4FET6	ATPUP3	−2.28		
GRMZM2G026523_T01	B4FQ14	Peptide transporter PTR2	−2.30		
GRMZM2G167758_T01	Q9FMC7	Nuclear transport factor 2 (NTF2) family protein	−2.32		
AC185254.4_FGT002	Q6ZIV9	Putative ABC transporter	−2.39		
GRMZM2G311401_T01	A0A096T5V7	Uncharacterized protein	−2.48		
GRMZM2G027891_T01	Q0JB23	Os04g0561000 protein	−2.54		
GRMZM2G091478_T01	K7TVU7	Uncharacterized protein	−2.68		
GRMZM2G036631_T01	K7V7R6	Uncharacterized protein	−2.69		
AC235544.1_FGT004	Q2QP91	Dor1-like family protein, expressed	−2.75		
GRMZM2G040871_T01	B6SKF6	Hexose transporter	−2.77		
GRMZM2G139639_T01	B6TJ37	Inorganic phosphate transporter 1-5	−2.78		2.72
GRMZM2G319781_T01	M8CDC8	Phosphatidylinositol transfer protein 2	−2.85		
GRMZM2G348726_T01	B4FJ28	Signal recognition particle 9 kDa protein	−2.93	−2.73	
GRMZM2G137108_T01	Q9ATN2	Aquaporin NIP2-2	−3.02		
GRMZM2G154845_T01	A0A096SLG9	Protein DETOXIFICATION	−3.19		−2.31
GRMZM2G168439_T01	B4F9E1	Aquaporin TIP1-2	−3.49	−3.14	
GRMZM2G168365_T01	B6T9U6	Bidirectional sugar transporter SWEET	−3.57		
GRMZM2G010779_T01	B6U903	Vacuolar cation/proton exchanger 2	−3.60	−2.44	
GRMZM2G351347_T01	C4J1B8	Calcium-activated outward-rectifying potassium channel 1	−3.67		
GRMZM2G305446_T01	Q9ATL7	Aquaporin TIP3-1	−3.70		
GRMZM2G144581_T01	A0A096SEB4	Bidirectional sugar transporter SWEET4	−4.01		
GRMZM2G060742_T01	B6SSI8	Citrate transporter family protein	−4.44	−2.55	
GRMZM2G101958_T01	B6SGP7	Non-specific lipid-transfer protein		7.01	
GRMZM2G037229_T01	A0A096QDL2	Probable magnesium transporter		5.44	
GRMZM2G107239_T01	Q0IQZ4	Os11g0695900 protein		4.87	
GRMZM2G173669_T01	B4FTL9	Sugar transporter SWEET		3.38	
GRMZM2G476069_T01	M7ZN84	Nitrate transporter 1.4		3.01	
GRMZM2G098088_T01	K7UTZ0	Hexose transporter		2.75	
GRMZM2G384661_T01	K7WBE5	Uncharacterized protein		2.71	
GRMZM2G425683_T01	B6TCP1	Carbohydrate transporter/sugar porter/transporter		2.37	
GRMZM2G152555_T01	W9R1V3	Oligopeptide transporter 1		2.28	
GRMZM2G092780_T01	C0P4H8	Phosphate transporter		2.22	
GRMZM2G130454_T01	B6UDW9	Lipid transfer protein		2.07	
GRMZM2G005293_T01	B6U0S4	Patellin-5		−2.06	
GRMZM2G150468_T01	Q0J9C7	Os04g0660900 protein		−2.09	
GRMZM2G093276_T01	E3WCP2	Zinc transporter		−2.32	
GRMZM2G047762_T01	A0A096QKI7	Uncharacterized protein		−2.32	
GRMZM2G009045_T01	Q67UA2	Putative phosphate transport protein, mitochondrial		−2.46	
GRMZM2G477872_T01	AB45G	ABC transporter G family member 45		−2.70	
GRMZM2G075150_T01	Q7XEN0	Exocyst complex component Sec15		−2.71	
GRMZM2G335218_T01	K7V706	Ammonium transporter		−2.80	−4.26
GRMZM2G055545_T01	A0A096QQK0	Uncharacterized protein		−2.88	
GRMZM2G347457_T01	B6TSV4	Peptide transporter PTR2		−3.47	
GRMZM2G070500_T01	B6TIX8	Nodulin-like protein			3.62
GRMZM2G061495_T01	Q7XMZ2	OSJNBa0027G07.3 protein			3.20
GRMZM5G865543_T01	B6SUB5	Electron carrier/electron transporter/iron ion binding protein			3.16
GRMZM2G476069_T01	M8C905	Nitrate/chlorate transporter			2.89
GRMZM2G057611_T01	Q67VA9	Putative oligopeptide transporter			2.85
GRMZM2G423884_T01	K7V9U9	Protein detoxification			2.79
GRMZM2G519761_T01	K7UHM7	Uncharacterized protein			2.64
GRMZM2G055834_T01	Q852B2	Os03g0823500 protein			2.64
GRMZM2G020859_T01	B6SV43	Potassium channel AKT2/3			2.36
GRMZM2G153961_T01	M7YVY5	ABC transporter B family member 11			2.34
GRMZM5G806774_T01	K3XV44	Glutamate receptor			2.23
GRMZM2G032899_T01	K7V706	Ammonium transporter			2.20
GRMZM2G011636_T01	A0A096PXD7	25.3 kDa vesicle transport protein			−2.12
GRMZM5G850455_T01	B6T2C9	Lipid transfer protein			−2.15
GRMZM2G015767_T01	B6U0T7	Peptide transporter PTR2			−2.21
AC234152.1_FGT007	K7UVH5	Potassium channel2			−2.28
AC203966.5_FGT006	M8CDC1	25.3 kDa vesicle transport protein			−5.56

*Genome ID, Maize transcript ID (ZmB73_5b_FGS_cdna.fasta.gz); FC, fold change value*.

Aa and Bio treatments caused also different modulations of transcripts involved in the uptake of N inorganic forms (${\text{NO}}_{3}^{-}$ and ${\text{NH}}_{4}^{+}$). Aa up-regulated (Aa vs. N) the ZmNRT2.2 (*GRMZM2G010251_T01*; Plett et al., 2010), a well-known gene involved in the inducible high affinity transport systems (iHATS) in maize roots (Garnett et al., 2013; Zamboni et al., 2014; Pii et al., 2016). Both Aa and Bio treatments down-regulated the ZmNRT1.2 transcript encoding a low affinity ${\text{NO}}_{3}^{-}$ transporter (*GRMZM2G137421_T01*; Garnett et al., 2013), while the expression of another low affinity ${\text{NO}}_{3}^{-}$ transporter, ZmNRT1.4B (*GRMZM2G476069_T01*) which is very low expressed during plant development (Garnett et al., 2013), seems to be Bio-specific (up-regulated both in Bio vs. Aa and Bio vs. N). Another Bio-specific transcript encodes a putative ${\text{NH}}_{4}^{+}$ transporter (AMT2; *GRMZM2G335218_T01*, down-regulated both in Bio vs. Aa and Bio vs. N).

Our analysis showed that transcripts involved in the uptake systems of other mineral nutrients were selectively affected by Aa and Bio. Organic N sources stimulated the expression of transcripts encoding putative yellow stripe-like (YSL) transporters (*GRMZM2G156599_T01, GRMZM2G024196_T01*, and *GRMZM2G135291_T01*) involved into the uptake of iron-phytosiderophore and distribution of metals within the whole plant (Curie et al., 2009). ZmYS1 (*GRMZM2G156599_T01*) encodes a protein that acts as a proton-coupled symporter of metals chelated to phytosiderophore and to nicotianamine (Schaaf et al., 2004) playing a key role in Strategy II of Fe acquisition utilized by the graminaceous species such as maize (Kobayashi and Nishizawa, 2012). A noticeable difference in the responses to organic N forms relies in the behavior of genes involved in nicotianamine and phytosiderophore biosynthesis. In particular, only in the Aa vs. N comparison we could observe a positive modulation of transcripts involved into nicotianamine synthesis (nicotianamine synthase, NAS; *GRMZM2G030036_T01, AC233955.1_FGT003, GRMZM2G124785_T01, GRMZM2G034956_T01, GRMZM2G385200_T01, GRMZM2G312481_T01*) and into phytosiderophore biosynthesis (deoxymugineic acid synthase, DMAS; *GRMZM2G060952_T01*) (Kobayashi and Nishizawa, 2012). However, in the comparison Bio vs. N only one gene related to phytosiderophore biosynthesis (nicotianamine aminotransferase, NAAT; *GRMZM2G096958_T01*) was found up-regulated. Taken together, even with defined differences these results indicate a strong impact of Aa and Bio with respect to Fe-stress responses of maize roots. Furthermore, besides Fe, nicotianamine forms transport coordination complexes with divalent transition metal cations, i.e., Mn^2+^, Zn^2+^, Cu^2+^ (Benes et al., 1983) whose concentrations were increased in Bio-treated roots.

The category of “establishment of localization” grouped also transcripts involved in S, P, and K transport processes. We observed a down-regulation of a transcript for a sulfate transporter (SULTR; *GRMZM2G080178_T01*; Hawkesford, 2002) when we compared the transcriptional profile of Aa- with that of N-treated roots. Concerning P, transcripts encoding putative Pi transporters (PHT; Raghothama, 2000) were down-regulated by Bio (*GRMZM2G009045_T01*) and Aa treatment (*GRMZM2G139639_T01*). Only in the comparison Bio vs. Aa two transcripts encoding for K channel AKT (Hirsch et al., 1998) were up- (*GRMZM2G020859_T01*) and down-regulated (*AC234152.1_FGT007*), respectively. Due to the presence of several members belonging to transporter families that have specific roles in nutrient uptake and translocation, and considering that transporter proteins are subjected to multiple forms of regulation (e.g., post-translational modifications), the transcriptional data do not allow a full explanation of the observed changes in tissue nutrient concentrations displayed by the different treatments.

Interestingly, a glutamate receptor (*GRMZM5G806774_T01*) functioning as non-selective cation channel is induced in Bio vs. Aa. This receptor, regulated by a broad range of amino acids, is involved in different physiological processes such as C/N sensing, resistance against fungal infection, root growth and response to wounding (De Bortoli et al., 2016).

#### Hormonal metabolism and signaling

A number of genes related to hormonal metabolism and signaling displayed expression changes in Bio- and Aa-treated seedlings vs. seedlings supplied with N (Table 4). A set of 9 genes whose expression profiles distinguished the Bio and Aa treatment vs. N represented the signature of organic N. Three of these genes coding for gibberellin 3-beta-dioxygenase 1 (*GRMZM2G046669_T01*), gibberellin 2-oxidase (*GRMZM2G022679_T01*) and gibberellin-regulated protein 2 (*GRMZM2G164090_T01*), respectively were related to gibberellin action. Gibberellin 3-beta-dioxygenase which converts inactive gibberellins (GAs) in their active form was up-regulated, whereas gibberellin 2-oxidase, implicated in GAs deactivation, was down-regulated in both Bio vs. N and Aa vs. N, suggesting that organic N forms induced the increase of active GAs in the roots. The other 5 genes that characterized the organic N supply were all related to auxin signaling or transport and were down-regulated in both Aa vs. N and Bio vs. N.

**Table 4:** **Differentially expressed transcripts involved in hormonal metabolism**.

**Genome ID**	**UniProt ID**	**Description**	**Aa vs. N (FC)**	**Bio vs. N (FC)**	**Bio vs. Aa (FC)**
GRMZM2G046669_T01	M8AKK4	Gibberellin 3-beta-dioxygenase 1	4.77	3.07	
GRMZM2G035156_T01	Q0DUR2	Transcription factor ILI6	4.68		
GRMZM2G011463_T01	B4FC68	SAUR37-auxin-responsive SAUR family member	3.83		
GRMZM2G462883_T01	N1R055	Putative gibberellin receptor GID1L3	2.63		
GRMZM2G093173_T01	K7U964	WAT-1-related protein	2.32	2.12	
GRMZM2G034917_T01	N1R055	Putative gibberellin receptor GID1L3	2.10		
GRMZM2G301932_T01	B6TXN5	Gibberellin receptor GID1L2	2.08		
GRMZM2G012546_T01	M8BFB1	Putative gibberellin receptor GID1L3	2.07		
GRMZM2G422419_T01	A0A096TQ86	Uncharacterized protein; response to auxin	2.0		
GRMZM2G364328_T01	B6TBZ6	WAT1-related protein	−5.57		
GRMZM2G050997_T01	Q709Q5	Cytokinin oxidase 2	−4.48		
GRMZM2G164090_T01	B6TLZ8	Gibberellin-regulated protein 2	−4.33	−3.34	
GRMZM2G150688_T01	B6TWS8	Gibberellin-regulated protein 1	−4.0		
GRMZM2G030790_T01	B6TLX4	Jasmonate-induced protein	−3.18		
GRMZM2G702564_T01	K7V9I8	Cytokinin oxidase 3	−3.04		2.02
GRMZM2G173732_T01	A2Z6Z0	Protein BIG GRAIN 1-like	−2.98	−3.01	
GRMZM2G065230_T01	B7ZXT3	WAT-1 related protein	−2.75	−2.7	
GRMZM2G420812_T01	B6T2P5	SAUR31-auxin-responsive SAUR family member	−2.74	−2.49	
GRMZM2G117878_T01	B6STN8	Cytokinin-N-glucosyltransferase 1	−2.51		
GRMZM2G013448_T01	C0PEP2	1-aminocyclopropane-1-carboxylate oxidase 1	−2.50		
GRMZM2G025742_T01	I3RWV5	Auxin efflux carrier component	−2.49		
GRMZM2G062019_T01	B6TWT9	Gibberellin receptor GID1L2	−2.49		
GRMZM2G368591_T01	K7V647	WAT 1- related protein	−2.44	−3.26	
GRMZM2G107900_T01	A0A096RQH9	Uncharacterized protein; response to auxin	−2.39		
GRMZM2G171822_T01	Q2QM77	Protein kinase PINOID	−2.15		
GRMZM2G022679_T01	Q8S0S6	Gibberellin 2-oxidase	−2.10	−2.34	
GRMZM2G068701_T01	Q0D4Z6	Probable indole-3-acetic acid-amido synthetase GH3.8	−2.06		
GRMZM2G141473_T01	O23888	Indole-3-acetaldehyde oxidase	−2.0		
AC233864.1_FGT009	Q7XTN9	OSJNBa0093O08.8 protein; response to auxin	−11.81	−6.49	
GRMZM2G471931_T01	K7TM25	Cytokinin riboside 5′-monophosphate phosphoribohydrolase		2.38	
GRMZM2G136567_T01	K7VFP2	WAT-1 related protein		3.43	
GRMZM2G330012_T01	A0A0B4J3E8	Uncharacterized protein; response to auxin		3.64	
GRMZM2G001977_T01	B6UC04	Gibberellin receptor GID1L2		−2.05	
GRMZM2G053338_T01	B6U4E2	Indole-3-acetic acid-amido synthetase GH3.8		−2.11	
GRMZM2G050321_T01	B6SZU3	Jasmonate O-methyltransferase		−2.69	
GRMZM2G024131_T01	Q41819	Indole-3-acetate beta-glucosyltransferase		−4.34	
GRMZM2G155680_T01	A0A096SLZ9	Cytokinin riboside 5′-monophosphate phosphoribohydrolase			2.04
GRMZM2G384762_T01	A0A096S9W7	Auxin-responsive protein			16.7

*Genome ID, Maize transcript ID (ZmB73_5b_FGS_cdna.fasta.gz); FC, fold change value*.

A characteristic feature of the Bio treatment vs. Aa and N was the induction of genes (*GRMZM2G471931_T01* and *GRMZM2G155680_T01*) coding for the enzyme cytokinin (CK) riboside 5′- monophosphate phosphoribohydrolase that converts CK ribosides in free CK. The up-regulation of these genes would result in an increased release of CKs from conjugates. CKs play an important role in root response to N supply coordinating root growth and N availability in the soil (Kiba et al., 2011; Kiba and Krapp, 2016). CKs also interact with auxin in determining root growth and architecture (Mi et al., 2008; Pacifici et al., 2015; Schaller et al., 2015). A second characteristic feature of the response of root to Bio was the down-regulation of *GRMZM2G053338_T01* and *GRMZM2G024131_T01* genes coding for a indole-3-acetic acid-amido synthetase and indole-3-acetate beta-glucosyltransferase, respectively. As these enzymes mediate the formation of IAA conjugate, their down-regulation indicates that Bio-treated roots retains a higher level of active indol-3acetic acid (IAA). The *GRMZM2G050321_T01* gene coding for a jasmonate O-methyltransferase was also down-regulated in Bio vs. N. Jasmonate O-methyltransferase catalyzes the formation of volatile methyl jasmonate that plays different roles in plant development and stress-response. In particular, high level of methyl jasmonate inhibits root growth. For instance, maize jasmonic acid-deficient *opr7opr8* double-mutant showed much longer lateral roots compared with wild type (Yan et al., 2014), phenotype that resembles the root morphology of Bio-treated plants (Figure 1C).

The analyses of the differentially expressed genes of the Aa vs. N comparison revealed the strong involvement of the gibberellin signaling pathway in the free amino acids action. Four genes (*GRMZM2G012546_T01, GRMZM2G462883_T01, GRMZM2G034917_T01, GRMZM2G301932_T01*) coding for putative GA receptors were up-regulated and one (*GRMZM2G062019_T01*) down-regulated in Aa vs. N comparison. Another interesting feature was the down-regulation of two genes involved in auxin transport (*GRMZM2G025742_T01* and *GRMZM2G171822_T01*), the first coding for a component of an auxin efflux carrier and the second coding for a PINOID kinase that regulates the membrane localization of the auxin efflux transporters PIN (Christensen et al., 2000). One of the effects of the Aa treatment was also the restraint of IAA and CK catabolism as the results of the down regulation of a gene coding for indole-3-acetaldehyde oxidase (*GRMZM2G141473_T01*) and two genes (*GRMZM2G702564_T01* and *GRMZM2G050997_T01*) coding for a CK oxidase 3 and a cytokinin oxidase 2, respectively.

The growth and architecture of the root apparatus is the result of the action of several phytohormones as well as their interplay. Therefore, it is not surprising that the activity of Bio and Aa on the root apparatus leaded to modifications in the metabolism and signaling pathways of different phytohormones. However, although some transcriptional changes were common to both treatments (i.e., the changes in the expression of genes coding for enzymes involved in GA metabolism), differences in the involvement of hormones in Bio and Aa root growth promoting effects have emerged. For instance, CK release from conjugated and inhibition of IAA conjugation together with a lower synthesis of methyl JA appeared the main effects of Bio, whereas the activity of Aa seems to result principally from altered GA synthesis and signaling and restrain of CK and IAA degradation.

A few transcripts coding for CLE peptide hormones were down-regulated in the analyzed comparisons (*GRMZM2G468688_T01* in Bio vs. N and Aa vs. N, *GRMZM2G165836_T01* in Bio vs. A and *GRMZM2G466532_T01* in A vs. N) and one was up-regulated (*GRMZM2G114127_T01* in Aa vs. N). On the other hand, *GRMZM2G438840_T01*, coding for CLE receptor kinase CLAVATA1 (CLV1) was up-regulated in Bio vs. N and Aa vs. N.

CLE signaling peptides and CLV1 play a notable role in sensing N (Miyawaki et al., 2013). It has been shown that lateral roots stop growing under severe deficiency of N, while the expression of CLE peptides is induced (Araya et al., 2014). This regulation serves as a mechanism to prevent the expansion of the lateral root system into N-poor environments (Gruber et al., 2013). The *clv1* mutant exhibits progressive growth of lateral roots under N-deficient conditions (Wang et al., 2016).

Interestingly, the transcripts *GRMZM2G055607_T01*, coding for a sulfotransferase which catalyzes post-translational tyrosine sulfation of secreted peptides, was over-expressed in the Bio vs. Aa comparison. In *Arabidopsis*, the loss-of-function mutant for tyrosyl-protein sulfotransferase, shows a short-root phenotype (Shinohara et al., 2016). Thus, this finding may represent another clue to unravel the action mechanisms of root growth stimulation exerted by Bio.

## Conclusions

The present study dissected the biostimulatory activity of short peptides and free amino acids on maize seedlings. We demonstrated that protein hydrolysates containing peptides and a very low fraction of free amino acids were more efficient in stimulating root growth and micronutrient accumulation than free amino acid mixture with the same amino acid composition, suggesting a specific role for small peptides in controlling root growth. The genome-wide transcriptional analysis of maize root responses to Bio and Aa as compared with inorganic N, allowed to shed light on the similarities and differences in the mechanisms of action of the two biostimulants. The Aa produced a stronger modification of transcriptional networks than Bio, 995 and 587 differentially expressed genes were detected in Aa vs. N and Bio vs. N, respectively. Both treatments displayed effects on genes related to oligopeptide and induced modifications of genes involved in ${\text{NO}}_{3}^{-}$ transport, demonstrating that N organic forms can interfere with inorganic N uptake, although the root total N was unchanged. On the other hand, a specific action of Bio seemed to be related to the regulation of the glutamate receptor which is involved in root growth and C/N signaling. Modification in genes implicated in metal ion transport were also detected in both treatments, although with some distinctive features. Even if plants were grown in the presence of Fe-EDTA and the content of Fe in the roots was unmodified, Aa and Bio positively affected components involved in Strategy II responses to Fe deficiency. In particular, Aa treatment caused the up-regulation of several transcripts involved in the synthesis of metal chelators (nicotianamine and mugineic acids) and in their transport. On the contrary, only three genes (one related to phytosiderophore synthesis and two to metal-phytosiderophore uptake and translocation) were up-regulated in Bio-treated roots. We might hypothesize that peptides could chelate metals facilitating their uptake and making in turn the biosynthesis of phytosiderophores less crucial. This might explain the higher contents of Cu, Mn, and Zn detected in maize roots treated with Bio. The stimulation of root growth was associated as expected, with perturbations in hormone balance. Both biostimulants modulated genes involved in GAs metabolism thus likely leading to increased GAs levels, and in auxin signaling and transport. In addition, Bio specifically modulated CKs release from conjugates and jasmonate metabolism. Future investigations aiming at studying the effects of protein hydrolysate and free amino acid applications on the content and distribuition of phytohormones in the plant, would be useful to deepen these findings. Noticeably, Aa treatment modified the expression of a high number of genes involved in the response to oxidative stress, whereas Bio caused only a modest modulation of stress-related genes. This might suggest that the lower growth promoting capacity of Aa respect to Bio might also be linked to the different metabolic engagement in stress responses.

## Author contributions

ZV and TP designed the experimental set up. CS and AZ performed the experiments. CS, AZ, ZV, and TP carried out the analysis of the data and draft the manuscript. TP coordinated the project. All the authors approved the manuscript.

### Conflict of interest statement

The authors declare that the research was conducted in the absence of any commercial or financial relationships that could be construed as a potential conflict of interest.
