# Supplementary material for: Growth Stimulatory Effects and Genome-Wide Transcriptional Changes Produced by Protein Hydrolysates in Maize Seedlings
Source: Front Plant Sci. 2017 Mar 30;8:433. doi: 10.3389/fpls.2017.00433 (PMC5371660; doi:10.3389/fpls.2017.00433)
Supplement: Supplementary file 1 [file Table1.DOC]

Probe ID, gene product and list of primers sequence for qRT-PCR analysis.

| **Transcript ID** | **Gene product** | **Product size** | **Primer sequences (forward/reverse)** |
| --- | --- | --- | --- |
| *GRMZM2G027378_T01* | *Ubiquitin-conjugating enzyme* | 267 | TGCGTTAATCACGAGACAGG  AATCACAAAGACAGGCAGGG |
| *GRMZM2G347457_T01* | *Peptide transporter* | 196 | CAATCGGCGTTGCCATTACT  TGAGCCCGCTGTTGATTTACT |
| *GRMZM2G096958_T01* | *Nicotianamine aminotransferase1* | 158 | TCAAATCTTTCTGCCAAAGGC  CTGCGTATTCAAGGAAAACT |
| *GRMZM2G429955_T01* | *Chlorophyll a-b binding protein 2* | 153 | CAAGTGAGCGAGGGCATGAT TATTGTTCAGCTCCACAGCAT |
| *GRMZM2G030036_T01* | *Nicotianamine synthase 2* | 180 | CGCTGTTTCAATCTCTCGTGT  GGTGCATTGTGCACACTGTTC |
| *GRMZM2G024996_T01* | *Glycine-rich cell wall structural protein* | 114 | CTGCGTAGCGAATAGCGATCA CTTGCTACCAACGCAGTGTAT |
