# Supplementary material for: Growth Stimulatory Effects and Genome-Wide Transcriptional Changes Produced by Protein Hydrolysates in Maize Seedlings
Source: Front Plant Sci. 2017 Mar 30;8:433. doi: 10.3389/fpls.2017.00433 (PMC5371660; doi:10.3389/fpls.2017.00433)
Supplement: Supplementary file 2 [file Table2.doc]

Composition of the protein hydrolysate

| **Protein Hydrolysate Composition** | **% (w/w)** |
| --- | --- |
| Total nitrogen | 11.3 |
| Total carbon | 30.0 |
| Free aminoacids | 10.0 |
| Total aminoacids | 62.5 |
| **Aminoacid profile** |  |
| Alanine | 7.0 |
| Arginine | 0.9 |
| Aspartic Acid | 3.0 |
| Glutamic Acid | 6.4 |
| Glycine | 15.8 |
| Hydroxylysine | 1.2 |
| Hydroxyproline | 5.3 |
| Histidine | 0.3 |
| Isoleucine | 1.1 |
| Leucine | 2.0 |
| Lysine | 2.1 |
| Methionine | 0.7 |
| Ornithine | 3.0 |
| Phenylalanine | 1.3 |
| Proline | 8.8 |
| Serine | 0.2 |
| Threonine | 0.3 |
| Tyrosine | 1.3 |
| Valine | 1.8 |
