# Supplementary figures and images for: Growth Stimulatory Effects and Genome-Wide Transcriptional Changes Produced by Protein Hydrolysates in Maize Seedlings
Source: Front Plant Sci. 2017 Mar 30;8:433. doi: 10.3389/fpls.2017.00433 (PMC5371660; doi:10.3389/fpls.2017.00433)

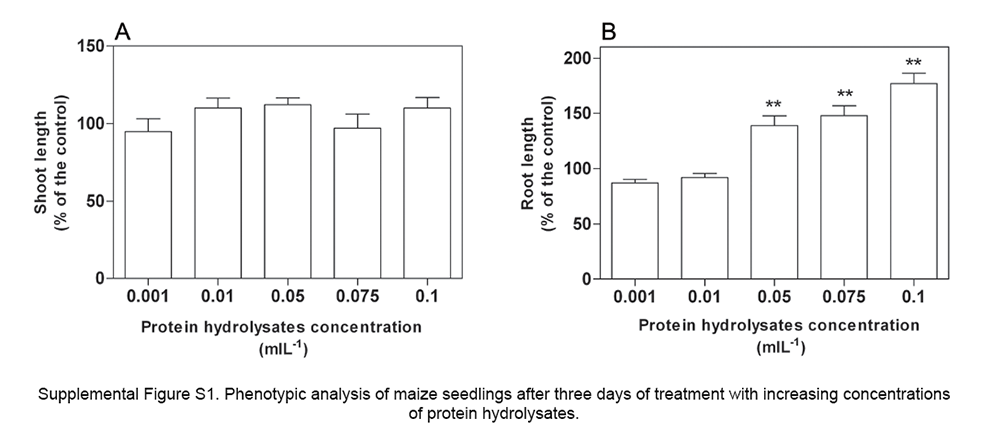

Supplement: Supplementary file 6 [file Image1.TIF]

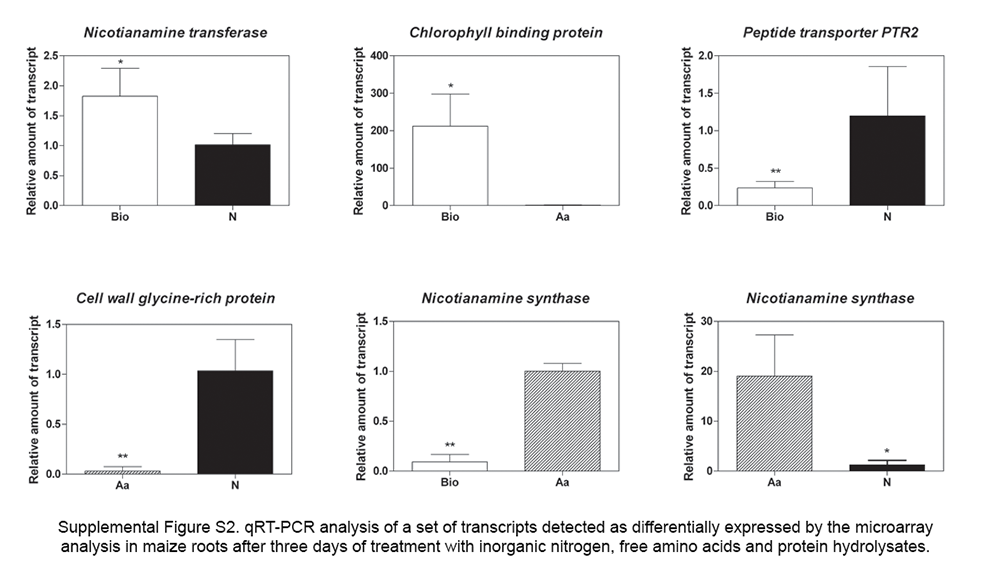

Supplement: Supplementary file 7 [file Image2.TIF]
